# Preoperative Single-Dose Intravenous Iron Formulation to Reduce Postsurgical Complications in Patients Undergoing Major Abdominal Surgery: A Randomized Control Trial Feasibility Study (PIRCAS Trial Pilot)

**DOI:** 10.7759/cureus.17357

**Published:** 2021-08-21

**Authors:** Thiri Naing Thin, Brenda Pei Yi Tan, Eileen Y Sim, Koin Lon Shum, Hazel Su Pin Chan, Hairil Rizal Abdullah

**Affiliations:** 1 Department of Anesthesiology, Singapore General Hospital, Singapore, SGP; 2 Department of Internal Medicine, Singapore General Hospital, Singapore, SGP; 3 Anesthesiology, Duke-National University of Singapore (NUS) Medical School, Singapore, SGP

**Keywords:** intravenous iron, perioperative medicine, blood transfusion, iron deficiency anemia (ida), pre operative anemia

## Abstract

Background

Preoperative anemia is associated with an increased need for blood transfusion, complications, and prolonged hospital stay. Iron deficiency anemia (IDA) may be treated with oral or intravenous (IV) iron. IV iron repletes iron stores more rapidly. Its impact on perioperative blood transfusion, postoperative complications, patients’ recovery, and long-term quality of life is unclear. Newer agents, such as ferric carboxymaltose (FCM), are costly but have higher maximum approved doses and a very low incidence of anaphylactic-type reactions. This study aims to explore the feasibility of a randomized control trial to compare the preoperative treatment of IDA with IV FCM versus oral ferrous fumarate, in patients undergoing elective major abdominal surgery.

Experimental design

This is an open-label pilot randomized controlled trial. A total of 30 adults with IDA scheduled for elective major abdominal surgery were recruited for the study. They were randomized into two groups to receive either oral iron or IV FCM. Primary outcomes are defined as the time from enrollment to study drug administration, recruitment rate, and follow-up rate up to three months. Secondary outcomes are hemoglobin rise from recruitment to surgery, perioperative blood transfusion, postoperative complications, EQ-5D-3L scores at baseline, and three months and adverse events related to IV FCM therapy.

Results

All patients received study drugs within five days of enrollment; 30 patients were recruited within four months, 15 patients in each group. Two in each group were withdrawn for surgery postponement. All patients were followed up for three months and there was no crossover of patients. Per protocol, analysis was performed. No severe adverse events related to IV FCM therapy occurred. Both groups had similar baseline characteristics, similar hemoglobin rise from enrollment to the day of surgery [0.2 (+1.6) g/dL in the FCM group and 0.8 (+0.7) g/dL in the Oral Iron group, p=0.3] and similar mean units of perioperative blood transfused (recruitment to discharge) per patient [1.3 (+ 2.1) in the FCM group and 0.9 (+1.3) in the Oral Iron group, p=0.6]. Postoperatively, there was a similar hospital length of stay [11.5 (+13.6 days) in the FCM group and 9.0 (+9.8 days) in the Oral Iron group, p=0.6]; there were similar postoperative complications as reflected by the average Comprehensive Complication Index [12.8 (+19.6) in the FCM group and 22.6 (+30.7) in the Oral Iron group, p=0.3]; similar postoperative health-related quality of life as reflected by mean EQ-5D-3L scores at one month [70.4 (+21.8) in the FCM group and 84.5 (+12.1) in the Oral Iron group] and three months [80.0 (+18.4) in the FCM group and 85.9 (+10.7) in the Oral Iron group].

Conclusions

A full-scale randomized controlled trial to evaluate the effectiveness of preoperative IV FCM compared to oral iron in patients with IDA undergoing major abdominal surgery is feasible.

## Introduction

The prevalence of preoperative anemia, defined as hemoglobin level <13 g/dL in males and <12 g/dL in females [1], ranges between 25% in knee arthroplasties and 75% in colorectal malignancies [2-4]. Many studies have documented the negative impact of preoperative anemia on post-surgical outcomes. These include adverse events such as the increased risk for perioperative blood transfusion, higher 30-day and one-year mortality, prolonged length of hospital stay, and increased healthcare cost [2,5-8].

Iron deficiency is one of the most common underlying causes of preoperative anemia [9]. Preoperative iron-deficiency anemia (IDA) may be effectively treated prior to surgery with oral or intravenous (IV) routes of iron replacement [10]. Oral iron supplementation is the cheapest and easiest form of iron replacement. However, its bioavailability is poor [11] and is further reduced in various diseases [12] with suboptimal adherence to therapy [11,13]. Full iron repletion may take three to six months of oral therapy [12]. This timeline may not always be available prior to major surgery. An alternative is the administration of IV iron, which can replenish iron stores more rapidly and effect faster improvement in Hb level [14-15]. However, older formulations of IV iron were associated with a high incidence of serious adverse drug reactions such as hypersensitivity reactions [16]. Iron sucrose was associated with less anaphylactic-type reactions but had to be given in small maximum dosages of 200 mg for each infusion, thus requiring several small dose infusions to achieve the calculated iron deficit. Newer agents such as ferric carboxymaltose (FCM) have much higher maximum approved doses up to 15 mg/kg per infusion but not more than 1000 mg per dose. Common reported minor side effects include nausea (occurring in 2.9% of the subjects), followed by injection/infusion site reactions, hypophosphatemia, headache, flushing, dizziness, and hypertension. Uncommon side effects include myalgia, arthralgia, pyrexia, and chest pain while anaphylactoid reactions are rare (<0.1% incidence) [17-18].

To date, there are limited studies [10,19-23] with mixed conclusions on the superiority of preoperative IV iron supplementation in reducing the postoperative complications rate and perioperative blood transfusion as compared to oral iron. FCM is attractive, in particular, as it has much higher maximum approved doses and allows for a single total dose infusion. Patients undergoing major abdominal surgery have a higher prevalence of iron-deficiency anemia, and often experience intraoperative bleeding and receive a perioperative blood transfusion. There is a need to investigate if preoperative IV iron therapy compared to oral iron therapy is truly beneficial for these patients. We hypothesize that treating IDA with a single dose of IV FCM infusion preoperatively, as compared to oral ferrous fumarate, will reduce post-surgical complications and perioperative blood transfusion and improve postoperative health-related quality of life measures in patients undergoing major abdominal surgeries.

We designed a preliminary study to assess the feasibility of conducting a full-scale trial. The primary objectives of this feasibility study are to assess the feasibility of the study design, in terms of recruitment rates, procedure implementation barriers, and outcome data variability, which will be used for sample-size calculation of a full-scale trial. The study design would be deemed feasible to be conducted in our institution if at least 96.7% (29 out of 30) participants received the study drug within five days of enrollment; if there is sufficient patient volume, defined as recruitment rate of 30 participants over four months; and if there is minimal loss to follow-up rate, defined as 90% of enrolled participants completing study follow-up. The secondary objectives of this feasibility study are to ascertain the effect of IV FCM compared to oral iron on the 30-day incidence of postoperative complications, perioperative blood transfusion requirements, change in hemoglobin levels, length of hospital stay (LOS), and health-related quality of life measurement with the EuroQol 5 Dimension 3 Levels (EQ-5D-3L).

## Materials and methods

This is an open-label, parallel, pilot randomized controlled trial (RCT) named Preoperative Single-Dose Intravenous Iron Formulation to Reduce Postsurgical Complications in Patients Undergoing Major Abdominal Surgery: A Randomized Control Trial Feasibility Study (PIRCAS Trial Pilot). Approval from the SingHealth Institutional Review Board (Ref No. 2017/2005) and registration at the US Clinical Trials database (NCT03295851) were done before its initiation. The study was conducted between November 2017 and May 2018 at Singapore General Hospital. As per standard clinical protocol, patients undergoing surgery are routinely scheduled for preoperative assessment at the Preoperative Evaluation Clinic (PEC) one to four weeks prior to surgery, which is the same time period required for iron supplementation to produce an effective rise in Hb levels [22]. A research nurse was stationed at PEC to screen and identify eligible participants. The study’s inclusion criteria include adults aged 21 years and older with iron-deficiency anemia, scheduled for elective major abdominal surgery, presenting between one and four weeks of their planned surgery, and who can receive the study intervention at least seven days before the date of surgery. The full study inclusion criteria are listed in Table 1. Written informed consent was obtained by study team doctors prior to trial initiation.

**Table 1:** Inclusion and Exclusion Criteria g/dL, Grams per Decilitre; µg/L, Micrograms per Litre

Inclusion Criteria	
1.	Elective major abdominal surgery (Duration ≥ 2 hours Or blood loss ≥ 500 ml)
2.	Age ≥ 21 years old
3.	Preoperative assessment visit scheduled 1 - 4 weeks before surgery
4.	Anemic (Male hemoglobin <13.0 g/dL; Female hemoglobin <12.0 g/dL)
5.	Iron deficient (Serum Ferritin <100 µg/L Or Serum Ferritin 100 µg/L - 300 µg/L & Transferrin Saturation < 20%)
6.	Able to receive infusion 1 - 4 weeks (at least 7 days) before the planned operation date
Exclusion Criteria	
1.	Known history of acquired iron overload
2.	Family history of hemochromatosis or thalassemia or transferrin saturation (TSAT) >50%
3.	Treatment with erythropoietin in the previous 12 weeks
4.	Known hypersensitivity to study drugs
5.	Patients with severe asthma or severe allergy (requiring hospitalization within the last 12 months)
6.	Pregnancy
7.	Age < 21 years old
8.	Involvement in other investigational medicinal product trials within the previous 4 weeks prior to randomization that may impact the results of this trial

Experimental Design

Randomization was performed by the research nurse using an online system (http://rct.mui.ac.ir/q/) after recruitment. Neither the study participant, research nurse, nor outcome adjudicators were blinded, as they were required to perform and/or receive the study interventions. Participants were randomly assigned to control or intervention arm in a 1:1 ratio stratified by age (<70 years or ≥70 years), baseline hemoglobin level (<10 g/dL or ≥10.1 g/dL), surgical site (upper or lower abdominal surgery), and surgical approach (open or laparoscopic).

Patients in the intervention arm received a single dose of IV FCM. The dosing for FCM is based on the maximum dose of IV iron that can be safely given in a single infusion (15 mg/kg or up to 1000 mg). The drug is diluted in 250 ml of normal saline and administered as an infusion over 30 minutes, which follows the Singapore Health Science Authority’s approved manufacturer’s product guideline. After completion of infusion, participants were monitored for half an hour for signs and symptoms of adverse drug reactions, which were recorded and analyzed as adverse events. Participants were also contacted via telephone call weekly until surgery day and asked to report adverse events. Participants in the control arm were prescribed ferrous fumarate 200 mg twice daily, to be taken until one day before the surgery.

Study Measurement and Outcomes

Baseline demographic data, comorbidities, details of surgery, full blood count, and anemia panel (transferrin saturation, serum iron, total iron-binding capacity, serum ferritin, serum vitamin B12, and serum folate levels) results on the day of recruitment were recorded. On the day of surgery, full blood count and anemia panel (transferrin saturation, iron, total iron-binding capacity, serum ferritin, serum vitamin B12, and serum folate levels) were repeated for all trial participants. Additionally, adverse events (AEs) related to study intervention and perioperative outcomes were recorded. These included blood transfusion, 30-day complications, 30-day mortality, and health-related quality of life measurement.

Health-related quality of life questionnaire was assessed using the EQ-5D-3L [24] at baseline; and, via telephone interview, at one month and three months after the operation. The EQ-5D-3L questionnaire comprises five domains that are graded at three levels and a visual analog scale (VAS) with two endpoints: “0 = worst imaginable health state to 100 = best imaginable health state [24]. Thirty-day postoperative complications were compiled via medical record review, classified using the Clavien Dindo Complications scoring system, and summarized into a single numerical score using the Comprehensive Complication Index (CCI) score [25].

Sample Size

The sample size was selected based on the observation that at least 12 patients per group provide adequate precision regarding the estimated mean and variance to allow for future sample size calculations [26]. We aim to have 15 patients in each group. This is to account for a possible missing data rate or loss to follow-up rate of three per group.

Statistical Analysis

A per-protocol analysis was performed. This includes patients who were randomized, received trial drugs, and underwent surgery and excludes those who were withdrawn. Continuous variables were summarized using their mean and standard deviations and compared using a two-sample t-test between the treatment and control groups while non-normally distributed discrete variables were summarized with their median and interquartile range and compared using the Mann-Whitney U test. Categorical variables were reported as frequency (percentage) and the chi-square/Fisher’s exact test was used to investigate if there was any discrepancy in baseline characteristics and outcomes in the two arms. Statistical analyses were performed using IBM SPSS Statistics for Windows, version 24.0. (Armonk, NY: IBM Corp.).

## Results

**Figure 1:**
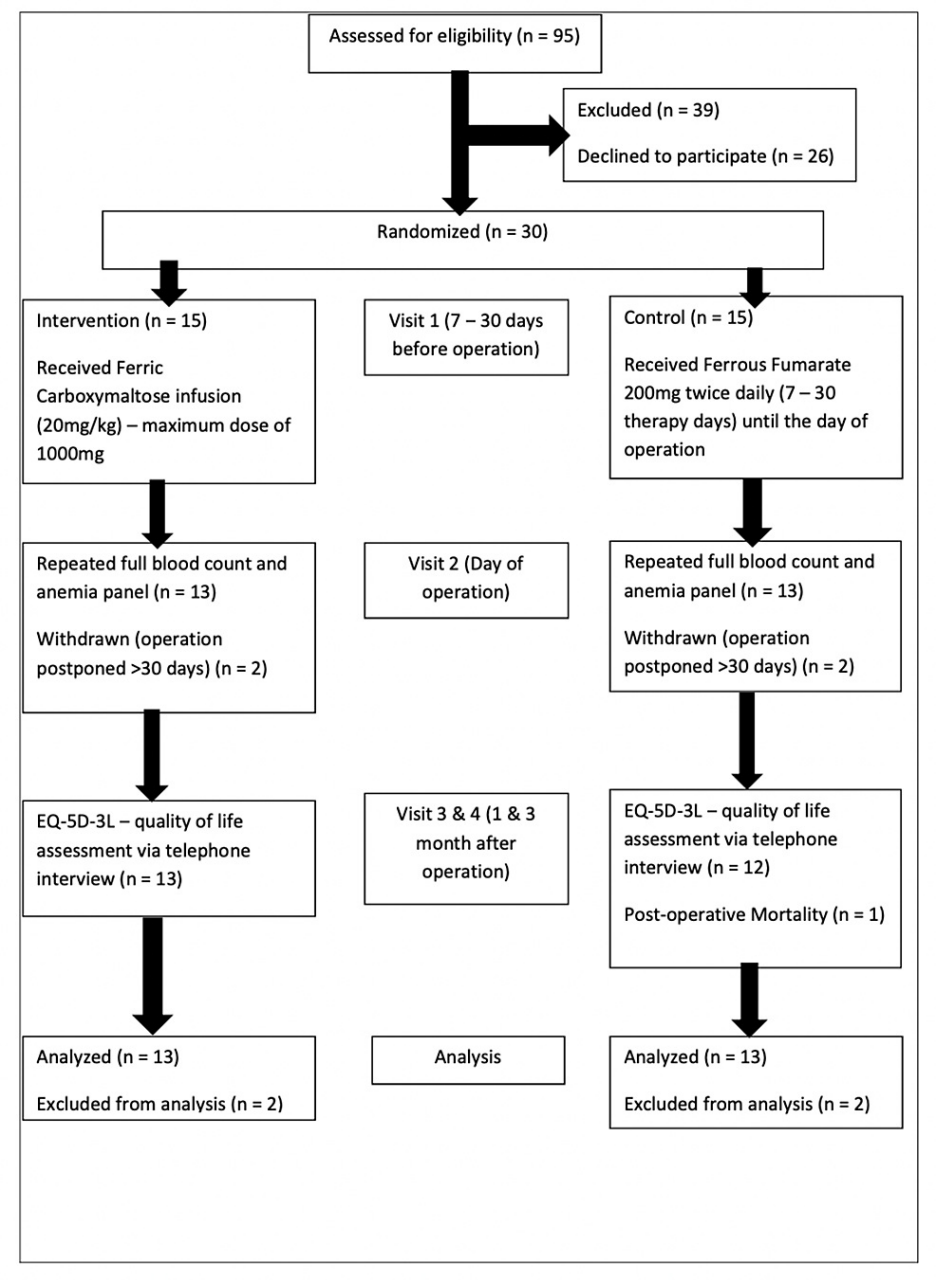
Trial Flowchart n, Number of Patients; mg, Milligram; kg, Kilogram

The primary aims of this feasibility study were time from recruitment to study intervention, recruitment rate, and the loss-to-follow-up rate. As shown in Figure 1, between November 2017 and May 2018, a total of 56 eligible patients were invited to participate in the trial. Twenty-six patients declined to participate mainly because they were not being able to choose the preferred study drug. The remaining 30 patients went through randomization, and 15 patients were allocated into the intervention and 15 into the control arm.

All patients received trial drugs within five days of enrollment. No severe adverse events related to IV FCM therapy occurred in the study. There was no crossover between the two treatment arms. Two participants from the IV FCM arm and two participants from the Oral Iron arm were withdrawn from the trial, as their surgeries were postponed >30 days and exceeded the trial’s allowable period from therapy to surgery. There was no loss to follow-up of the remaining 26 patients, and all completed the full study follow-up to three months.

Secondary Clinical Outcomes

The baseline characteristics of the two arms are shown in Table 2. Patients in the two arms had similar age, preoperative hemoglobin levels, preoperative indices of iron deficiency, such as serum ferritin, serum iron, and transferrin saturation levels; a similar proportion of gender, race, and equal distribution of surgical sites, and surgical approach. The only characteristic that differed significantly was the type of comorbidities as characterized by the Charlson Comorbidity Index (CCI). The median CCI score was significantly higher in the FCM group (6, IQR 2-7.5) compared to the oral iron group (2, IQR 0-3), p=0.03.

**Table 2:** Characteristics of the FCM and Oral Iron Group BMI, Body Mass Index; Hb, Hemoglobin; IQR, Interquartile Range; N, Number of Patients; SD, Standard Deviation; g/dL, Grams per Decilitre

Characteristic	Ferric Carboxymaltose (IV Iron)	Ferrous Fumarate (Oral Iron)	P-value
	(N = 13)	(N = 13)	
Age in years - Mean (SD)	59.2 (12.4)	55.2 (23.3)	0.6
Sex			
Female (%)	10 (76.9)	8 (61.5)	0.4
Race (%)			
Chinese	12 (92.3)	11 (84.6)	0.6
Malay	0	1 (7.7)	
Indian	1 (7.7)	1 (7.7)	
Charlson Comorbidity Index - Median (IQR)	6 (2,7.5)	2 (0,3)	0.03
Age-adjusted Charlson Comorbidity Index - Median (IQR)	7 (2.5,8)	3 (0,6)	0.08
BMI - Mean (SD)	24.6 (5.9)	25.5 (8.2)	0.8
Preop Hb in g/dL - Mean (SD)	10.8 (1.4)	10.2 (1.3)	0.3
Days between therapy and surgery - Mean (SD)	11.5 (3.0)	11.1 (3.5)	0.7
Serum iron - Mean (SD)	8.8 (4.7)	7.0 (4.5)	0.3
Serum ferritin - Mean (SD)	37.1 (52.8)	31.3 (41.0)	0.8
Serum transferrin saturation - Mean (SD)	12.8 (7.2)	11.8 (8.9)	0.8
Surgical site			
Upper abdomen (%)	4 (30.8)	4 (30.8)	1
Lower abdomen (%)	9 (69.2)	9 (69.2)	
Surgical Approach			
Open (%)	5 (58.5)	5 (38.5)	1
Laparoscopic (%)	8 (61.5)	8 (61.5)	

There was no significant difference in days between therapy and surgery in both groups and was on average 11.3 +3.2 days. Within the FCM group, no patient received a preoperative transfusion while in the oral iron group, three patients did. Excluding the patients who received a preoperative blood transfusion, the mean hemoglobin rise was 0.2 (+ 1.6) g/dL in the FCM group and was 0.8 (+ 0.7) g/dL in the oral iron group. Significantly more patients in the FCM group received intraoperative transfusion (6/13; 46.2%) compared to the oral iron group (1/13; 7.7%), p=0.03. Overall, from recruitment to discharge, the proportion of patients who received blood transfusion was identical in both groups - 6/13 (46.2%), and the mean units of blood transfused per patient was similar - 1.3 (+ 2.1) in the FCM group and 0.9 (+ 1.3) in the Oral Iron group. Further details can be seen in Table 3.

**Table 3:** Hemoglobin Improvement and Blood Transfusion Outcomes Between the FCM and Oral Iron Groups RBC, Red Blood Cells; N, Number; S.D, Standard Deviation; g/dL, Grams per Decilitre

Outcomes	Ferric Carboxymaltose (IV Iron)	Ferrous Fumarate (Oral Iron)	P-value
	(N = 13)	(N = 13)	
Mean rise in Hemoglobin in g/dL * - mean ( S.D.)	0.2 (1.6)	0.8 (0.7)	0.3
Number of patients who received RBC transfusion from recruitment to discharge – N (% within the group)	6 (46.2)	6 (46.2)	1
Any RBC transfusion from recruitment to discharge – N (% within the group)	6 (46.2)	6 (46.2)	1
Mean RBC units per patient in each group from recruitment to discharge - mean ( S.D.)	1.3 (2.1)	0.9 (1.3)	0.6
Mean RBC units per transfused patient in each group from recruitment to discharge - mean ( S.D.)	2.8 (2.3)	2.0 (1.3)	0.5
Number of patients who received RBC transfusion preoperatively – N (% within the group)	0 (0)	3 (23.1)	0.07
Mean RBC units transfused per patient preoperatively – mean ( S.D.)	0	0.4 (0.9)	0.1
Number of patients who received RBC transfusion intraoperatively – N (% within the group)	6 (46.2)	1 (7.7)	0.03
Mean RBC units transfused intraoperatively- mean ( S.D.)	0.9 (1.3)	0.2 (0.6)	0.06
Number of patients who received RBC transfusion postoperatively – N (% within the group)	2 (15.4)	4 (30.8)	0.4
Mean RBC units transfused postoperatively - mean ( S.D.)	0.4 (1.0)	0.4 (0.7)	1
* Exclude patients who received RBC transfusion			

The average hospital length of stay was slightly higher in the FCM group (11.5 + 13.6 days) as compared to the Oral group (9.0 + 9.8 days) (Table 4). While the proportion of patients in each arm who have more serious Clavien Dindo complications (grade 3a and above) are roughly similar - 4/13 in the FCM group and 6/13 in the Oral Iron group, there was one perioperative 30-day mortality in the Oral Iron group. Thus, the average comprehensive complication index is lower in the FCM group (12.8 + 19.6) as compared to the Oral Iron group (22.6 + 30.7). 

**Table 4:** Postoperative Complications and Quality of Life Between the FCM and Oral Iron Groups CCI, Comprehensive Complication Index; DAOH, Days Alive and Out of Hospital; EQ-5D-3L, EuroQol 5 Dimension 3 Levels; FCM, Ferric Carboxymaltose; FFP, Fresh Frozen Plasma; N, Number of patients; POD, Postoperative Day; POMS, Postoperative Morbidity Survey; RBC, Red Blood Cell; SD, Standard Deviation; VAS, Visual Analog Scale * 4 out of 12 as one patient had 30-day mortality

Outcomes	Ferric Carboxymaltose (IV Iron)		Ferrous Fumarate (Oral Iron)	P-value	
	(N = 13)		(N = 13)		
30-day CCI Score - Mean (SD)	12.8 (19.6)		22.6 (30.7)		0.3
Clavien Dindo Score 1&2	4 (33.3%)		4 (33.3%)		
Clavien Dindo Score 3a - 5	2 (16.7%)		3 (25%)		
Clavien Dindo Score 3	2 (16.7%)		1 (8.3%)		
Clavien Dindo Score 4	0		1(8.3%)		
Clavien Dindo Score 5	0		1 (8.3%)		
Total POMS score >= 1 at POD3	10 (66.7%)		9 (60%)		0.7
Total POMS score >= 1 at POD5	6 (40%)		4 (26.7%)		0.4
Total POMS score >= 1 at POD7/8	4 (26.7%)		5 (33.3%)		0.7
Total POMS score >= 1 at POD14/15	2 (13.3%)		3 (20%)		0.6
Total POMS score >= 1 at POD21/22	1 (6.7%)		1 (6.7%)		1
Total POMS score >= 1 at POD28/29	1 (6.7%)		0		0.3
Length of stay in days - Mean (SD)	11.5 (13.6)		9.0 (9.8)		0.6
Outcomes	Ferric Carboxymaltose (IV Iron)	N	Ferrous Fumarate (Oral Iron	N	P-value
6-month readmissions	4 (30.8%)	13	4 (33.3%) *	12	0.9
Total DAOH within 30 days - Mean (SD)	19.3 (8.9)	13	18.6 (10.2)	12	0.9
Total DAOH within 3 months - Mean (SD)	75.2 (16.1)	13	68.8 (26.9)	12	0.5
Total DAOH within 6 months - Mean (SD)	166.8 (14.5)	13	270.7 (442.0)	12	0.4
EQ-5D-3L (Health-Related Quality of Life) - (VAS score) Baseline - Mean (SD)	70.3 (22.0)	15	73 (8)	15	0.6
EQ-5D-3L (Health-Related Quality of Life) - (VAS score) 1 month - Mean (SD)	70.4 (21.8)	13	84.5 (12.1)	11	0.07
EQ-5D-3L (Health-Related Quality of Life) - (VAS score) 3 month - Mean (SD)	80.0 (18.4)	13	85.9 (10.7)	11	0.4

The proportion of patients who were readmitted within six months of surgery was identical at 4/13 in each group. DAOH is a patient-centric composite outcome that integrates clinically important outcomes of death, hospital length-of-stay, and multiple, if any, hospital readmission(s) [27]. It reflects a patient’s experience after surgical intervention. In our study, while both arms had largely similar DAOH at 30 days, the gap widened at three months (75.2 + 16.1 days in FCM, 68.8 + 26.9 days in the Oral Iron group) and even further at six months (166.8 + 14.5 days in FCM, 270.7 + 442.0 days in oral iron). These differences were not statistically significant due to the small numbers and large variations between patients (Table 4).

## Discussion

The primary aim of this PIRCAS Trial Pilot study is to test the feasibility of conducting a full-scale, adequately powered randomized controlled trial comparing the impact of preoperative single-dose IV FCM versus oral iron replacement on perioperative outcomes in patients with iron deficiency anemia undergoing major abdominal surgery. It would be deemed feasible if 96.7% (29 out of 30) of the participants receive their allocated study drug within five days of enrollment; 30 participants were recruited within four months and there was complete follow-up of at least 90% of participants. We achieved all our primary objectives. Despite occasional reports of serious adverse reactions associated with IV FCM in literature, we did not observe any during the conduct of the trial [15,28].

The cutoff of four months as the maximum amount of time that should be taken to recruit 30 patients was chosen, as we estimated that a full-scale trial would require about 180-270 patients based on other full-scale studies [10,19,29]. Thus, a recruitment rate of 30 patients over four months may allow the full-scale trial to be completed within two to three years approximately. These findings suggest that a full-scale trial is feasible. However, towards the end of the pilot study, there was a concurrent initiative in our institution to introduce IV iron as a standard of care for severely anemic patients undergoing major surgery, hence patients and their physicians became more reluctant to enroll them into a randomized controlled trial. Our recruitment slowed as a consequence, although we were still able to meet our target. Hence, we feel that a full-scale trial can only be accomplished by expanding this into a multicenter trial and involving other local institutions where IV iron has not gained a foothold in routine clinical care.

The secondary aims of the study are to examine the impact of IV FCM on perioperative blood transfusion, postoperative complications, hospital length of stay, readmission, days alive and out of hospital, and health-related quality of life. This trial was not powered adequately to analyze for secondary outcomes, and the small sample size may introduce a Type II error if we attempt to draw conclusions about the outcomes [30]. Nevertheless, we observe no significant differences in mean hemoglobin rise from initiation of therapy to the day of surgery or overall blood transfusion from recruitment to discharge. Oral group participants had a higher average comprehensive complication index that was almost twice that of the FCM group, likely due to the mortality in the oral iron group. However, in spite of the higher perioperative complication index, the oral group fared better in terms of slightly shorter hospital length of stay, more days out of hospital in the ensuing six months after surgery, and better overall health-related quality of life at one month compared to the FCM group, although these results did not achieve statistical significance. A full-scale randomized controlled trial that is powered to examine these secondary outcomes is needed to confirm these findings.

Preoperative IV iron supplementation is hypothesized to effect a faster hemoglobin rise compared to oral iron that may reduce the need for blood transfusion. It takes on average two to four weeks to effect a 1 g/dL rise in hemoglobin after iron supplementation. However, our study result demonstrated that there is no difference in the rate of rise of hemoglobin levels on the day of surgery between both groups. We postulate that this is because our study is underpowered for this outcome, and because of the short time from therapy to surgery in our study (average 11.3 days).

Our results contrast with the results of another full-scale study by Keeler et. al that randomized anemic patients with non-metastatic colorectal adenocarcinoma to receive oral or intravenous FCM. They were able to demonstrate that the FCM group had significantly higher increases in hemoglobin after treatment (median 1⋅55 (i.q.r. 0⋅93-2⋅58) versus 0⋅50 (−0⋅13 to 1⋅33) g/dl; P < 0⋅001) compared to oral iron [10]. Notably, their average time to surgery was 20 days in the FCM group and 26.5 days in the oral iron group, which were longer than ours. However, in terms of overall transfusion from recruitment to discharge in both arms, there were no significant differences in the number of patients who received a blood transfusion in each arm or the mean units of blood transfused per patient [10].

The PREVENTT (preoperative intravenous iron to treat anaemia before major abdominal surgery) trial is the largest multi-center, double-blind, parallel-group, randomized study to date examining the impact of preoperative IV iron compared to placebo in treating anemia in adult patients before major open abdominal surgery. Likewise, similar to Keeler’s study, they found that while IV iron was able to effect a significant improvement in hemoglobin levels compared to baseline by the time of surgery (mean difference [MD] 4·7 g/L, 95% CI 2·7-6·8), there was no difference in blood transfusion in the two groups. However, the preoperative IV iron group had significantly higher mean hemoglobin values after surgery at eight weeks and six months compared to the baseline, and reduced hospital readmission for complications, despite no differences in the type of surgery, bleeding, or transfusion volumes between the group [23].

## Conclusions

We have demonstrated that it is feasible to conduct a full-scale, adequately powered randomized controlled trial comparing the impact of a single preoperative dose of IV FCM versus oral iron replacement on perioperative outcomes in patients with iron deficiency anemia undergoing major abdominal surgery in a single high-volume surgical center that does not routinely prescribe IV iron preoperatively for patients with severe iron-deficiency anemia. However, our recruitment rate slowed down towards the tail end of our study when the prescription of IV iron became part of routine care in our center. This posed a challenge for study recruitment, as patients were unwilling to undergo randomization since they already had their minds set on receiving IV iron.

Our pilot study was underpowered to demonstrate any significant differences in perioperative blood transfusion, hospital LOS or complication rates. A full-scale randomized controlled trial that examines these outcomes would need to be conducted in centers that do not have plans to prescribe IV iron routinely in the near future. In light of the publication of the PREVENTT trial, which has quite definitively answered the impact of IV iron on perioperative blood transfusion, subsequent trials on IV iron could consider focusing on the effect of preoperative IV iron and increased postoperative hemoglobin levels in reducing readmission to hospital for surgical complications.
